# Combining Genomic and Genealogical Information in a Reproducing Kernel Hilbert Spaces Regression Model for Genome-Enabled Predictions in Dairy Cattle

**DOI:** 10.1371/journal.pone.0093424

**Published:** 2014-03-26

**Authors:** Silvia Teresa Rodríguez-Ramilo, Luis Alberto García-Cortés, Óscar González-Recio

**Affiliations:** 1 Departamento de Mejora Genética Animal, Instituto Nacional de Investigación y Tecnología Agraria y Alimentaria (INIA), Madrid, Spain; 2 Departamento Técnico Conafe, Madrid, Spain; 3 Department of Environment and Primary Industries, Biosciences Research Division, Melbourne, Victoria, Australia; 4 Dairy Futures Cooperative Research Centre, Melbourne, Victoria, Australia; National Taiwan University, Taiwan

## Abstract

Genome-enhanced genotypic evaluations are becoming popular in several livestock species. For this purpose, the combination of the pedigree-based relationship matrix with a genomic similarities matrix between individuals is a common approach. However, the weight placed on each matrix has been so far established with *ad hoc* procedures, without formal estimation thereof. In addition, when using marker- and pedigree-based relationship matrices together, the resulting combined relationship matrix needs to be adjusted to the same scale in reference to the base population. This study proposes a semi-parametric Bayesian method for combining marker- and pedigree-based information on genome-enabled predictions. A kernel matrix from a reproducing kernel Hilbert spaces regression model was used to combine genomic and genealogical information in a semi-parametric scenario, avoiding inversion and adjustment complications. In addition, the weights on marker- *versus* pedigree-based information were inferred from a Bayesian model with Markov chain Monte Carlo. The proposed method was assessed involving a large number of SNPs and a large reference population. Five phenotypes, including production and type traits of dairy cattle were evaluated. The reliability of the genome-based predictions was assessed using the correlation, regression coefficient and mean squared error between the predicted and observed values. The results indicated that when a larger weight was given to the pedigree-based relationship matrix the correlation coefficient was lower than in situations where more weight was given to genomic information. Importantly, the posterior means of the inferred weight were near the maximum of 1. The behavior of the regression coefficient and the mean squared error was similar to the performance of the correlation, that is, more weight to the genomic information provided a regression coefficient closer to one and a smaller mean squared error. Our results also indicated a greater accuracy of genomic predictions when using a large reference population.

## Introduction

Genomic selection refers to artificial selection decisions made using breeding values predicted from dense marker data [1]. These predicted breeding values are usually called direct genomic values (DGV) when only marker information is used, or genomic estimated breeding values (GEBV) when they are combined with pedigree information, often *a posteriori* in a blending procedure, or resulting from a single step method [2]. Different approaches may be used as genome-enhanced prediction methods for predicting DGV. These can be based on regularized linear regression in marker models [1], [3] or on relationship matrices between individuals calculated using genomic information [4], [5], [6]. The latter is commonly known as the genomic best linear unbiased prediction (G-BLUP) method. Usually one assumes the same variance in all loci, treats them all as equally important, and builds a genomic relationship matrix (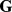) via genomic similarity between individuals.

Various 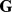 matrices have been proposed, with those in VanRaden [5] and Yang et al. [6] being the most commonly used in practice, differing in the manner in which allele frequencies of markers are handled. Several studies showed that G-BLUP performs as well as other models for many traits, especially for those where the expression of the phenotype is driven by many genes with a small effect each [7], [8].

Initially, a limitation to this approach was that the 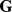 matrix may not be positive definite, *e. g*., if there are animals with identical genotypes (such as clones, monozygotic twins, or genotyping errors). In these cases, the unique inverse of the 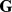 matrix, which is a pre-requisite for most available software for running G-BLUP, does not exist.

This problem has been overcome by blending 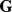 with the pedigree-based numerator relationship matrix (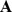), or even with an identity matrix (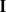); the former has been shown to provide more accurate predictions than the latter. An *ad hoc* positive weight (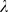) is used to form the linear combination 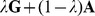. In addition, use of pedigree-based information, together with marker-based information, may improve genomic predictions because the SNP information may not account for all additive genetic variance, and it also allows utilizing data from genotyped and non-genotyped individuals together, as proposed in the single step method of Misztal et al. [9].

VanRaden [5] indicated that reliability (squared correlation) of predictions increased only by 0.0002 when varying 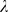 from 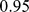
to 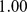. In addition, he showed that reliability was lower for 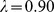 than for 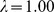. Aguilar et al. [10] and Forni et al. [11] found minor differences in DGV when using a weight between 0.95 and 0.98. Christensen and Lund [12] indicated that 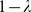 could be interpreted as the relative contribution of infinitesimal effects relative to the total additive variance, that is
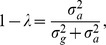
where 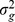 is the variance explained by the markers, and 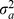 is the variance due to the infinitesimal effects.

The weight applied to each matrix should be carefully considered as it may depend on circumstances specific to each particular case, such as genotype density, amount of incorrect or missing pedigree available, genetic architecture and heritability of the trait, and number of animals and phenotypes used in the evaluation. Therefore, global recommendations on the weights assigned to 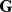 and 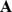 should be taken with care because these may not suit every population or trait. The choice of 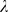 should be made using a proper statistical assessment of the weights applied to each matrix, instead of employing *ad hoc* weights based on small differences observed between so-called ‘sensible’ values of 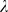.

Another important factor to take into account when combining marker- and pedigree-based relationship matrices is that marker- and pedigree-based relationship matrices may not be on the same scale. A straightforward reason is that allele frequencies in the base population are unknown in livestock or humans, and the adjustments proposed by VanRaden [5], Yang et al. [6] or Vitezica et al. [13] do not translate 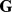 and 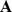 to the same base and scale [14].

These problems can be overcome by combining 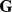 and 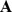 matrices with weights estimated properly. This study proposes a novel method for estimating the weights for 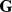 and 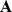 in a semi-parametric model using a Bayesian framework. The semi-parametric model is a Reproducing Kernel Hilbert spaces (RKHS) regression that allows combining 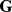 and 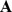 in a kernel matrix while making weaker assumptions on the compatibility of both matrices. The RKHS approach was first proposed by Gianola et al. [15] and has been implemented in real data by other authors [16]–[20]. The Bayesian framework allows flexible estimation of 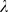 using samples from conditional distributions in MCMC algorithms.

This article is organized as follows: the first section, Reproducing kernel Hilbert spaces, reviews RKHS in a genomic selection context and discusses connections between RKHS and G-BLUP. The section on weighting factor estimation describes the novel approach for estimating the weight to be assigned to each matrix in a Bayesian RKHS framework. In the data analysis section, the proposed methodology is applied to a real data set representing a large number of genotyped animals. Concluding remarks are provided in the final section of the article.

## Materials and Methods

### Reproducing Kernel Hilbert Spaces Regression

Reproducing kernel Hilbert spaces regression [21] is a semi-parametric approach that allows the inference of a given function without making strong prior assumptions about functional form. In the context of genomic selection a function on genotypes of SNP markers is estimated to predict genomic-enhanced genotypic values or breeding values if the kernel encodes additive effects [4], [16].

This method assumes that distances in an Euclidean space can be represented via a kernel matrix reflecting distances between objects (focal points) in a Hilbert space. In our case, the non-parametric function is 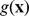, which is an unknown function of SNP markers 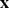. The semi-parametric model is
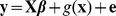
and the penalized sum of squares has the form [21], [22]:
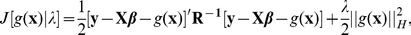
(1)where 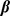 is a vector of nuisance parameters, 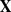 is an incidence matrix, 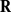 is the residual covariance matrix and 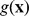 is as defined earlier. The second term in this equation acts as a penalty, adding up some deviance that depends on the value of an unknown regularization parameter 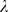. The term 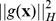 is a norm under a Hilbert space. The penalty can be made more complex if several kernels are fitted simultaneously [23].

Kimeldorf and Wahba [24] found that the function 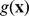 that minimizes (1) admits the following representation:
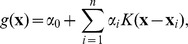
where 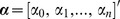 is a vector of unknown coefficients, and *n* is the number of observations (in our case training genotyped animals). In this implementation, the intercept 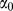 may be included in the model as a population mean or nuisance parameter. Further, 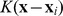 is a reproducing kernel used as basis function, possibly depending on some smoothing parameter(s) 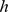. The 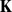 matrix may be based on any similarity function between individuals, *e. g*., in a genomic selection scenario it is any function of similarities based on markers. Here, the 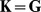 matrix meets the requisites of a kernel matrix, as it is positive semidefinite, defines genomic similarities between genotyped individuals and meets the distance requirements in a Hilbert space [25]. Hence, the non-parametric function can be expressed as 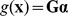.

Embedding this expression in (1) the function to be minimized becomes:
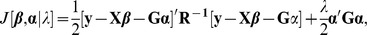
After setting the gradients with respect to 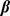 and 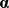 to 0 [4], [15], [26], the RKHS regression equations can be formulated in matrix form as:


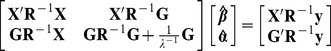
, or pre-multiplying the second subsystem by 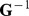 we obtain
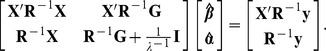
Equivalently, the RKHS approach can be formulated in terms of the random effects model:
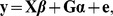
with the non-parametric coefficients (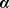) and the residuals (**e**) assumed to be independently distributed as 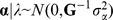 and 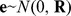, respectively, with 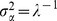 and 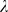 distributed as an uniform (0,1) random variable (the latter being a Bayesian assumption made here).

This may be viewed as a reparametrization of a G-BLUP model, as shown by De los Campos et al. [27] where the DGV (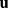) can be expressed as 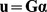. Both models are equivalent, leading to the same solutions, as shown in Table 1, when ignoring nuisance effects.

**Table 1:** Reparameterization of the Bayesian RKHS (reproducing kernel Hilbert spaces) and the G-BLUP. Adapted from De los Campos et al. [27].

	Bayesian RKHS Reparametrization I	G-BLUP Reparametrization II
Equations in the linear model	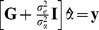	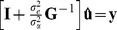
Breeding value prediction	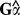	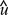
A priori distribution of the genomic effects	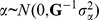	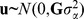

This reparametrization has two main advantages: (1) 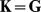 does not need to be inverted, and (2) prediction of genotyped animals without phenotypes can be done by multiplying the estimated non parametric coefficients times a rectangular supporting matrix 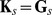 containing the genomic similarities (elements in the 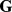 matrix) between individuals used to infer the non-parametric coefficients and the new individuals whose DGV are to be predicted as
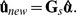
These two advantages offer a suitable framework for combining 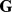 and 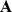 into a single kernel matrix, as described in the following section.

### Weighting factor estimation

Combining 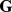 and 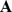 in a joint analysis poses some difficulties as stated previously. The semi-parametric RKHS is a suitable framework for overcoming some of the issues, as the combination of 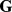 and 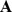 may be interpreted as a single kernel matrix in a scenario lacking parametric interpretation. The Bayesian approach allows estimation of the weight given to each matrix. These two matrices can be combined into a single kernel matrix 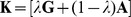, where 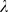 is the parameter to be estimated.

The model is as follows:
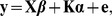
where 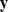 is the vector of phenotypic data (usually some sort of adjusted progeny performance for sires), 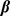 is the vector of nuisance variables assumed to be distributed as 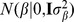, 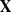 is an incidence matrix, 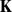 is the kernel matrix described above, 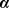 is the vector of non-parametric coefficients assumed to be distributed as 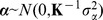, with 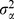 corresponding to the non-parametric variance and 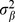 is large to produce a reasonably “flat” normal distribution. Here, residuals 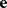 were assumed to be distributed as 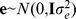, with 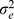 being the residual variance.

The implementation of a hierarchical Bayesian model for inferring model unknowns is described in Appendix S1.

**Figure 1:**
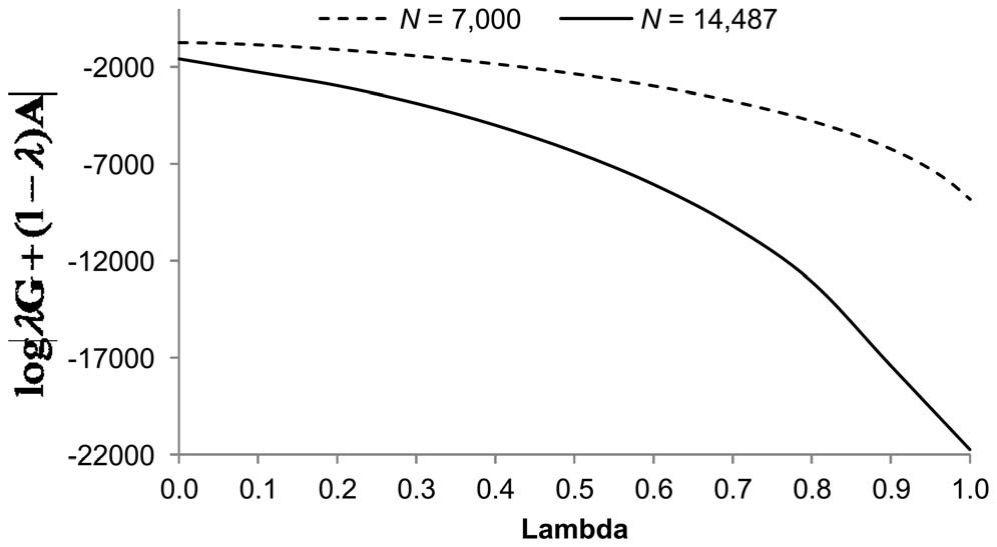
Approximated function of 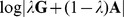 for different 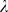 with *N* = 7,000 and *N* = 14,487.

To implement the inverse method for drawing 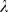 values from equation 1 (Appendix S1), the most computational intensive calculation is that of 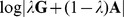. We overcame this by previously calculating this determinant over a grid of 101 values of 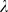 from 0 to 1 with increments of 0.01, and for 11 values of 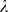 from 0 to 1 with increments of 0.1 for *N* = 7,000 and *N* = 14,487 individuals in the training data set, respectively (see details in the Data Analysis section). To set up the inverse approach, densities for each value of 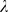 were obtained using increments of 0.0001. The 10,001 values of 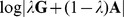 required for these evaluations were obtained by linear interpolation from the 101 or 11 explicitly calculated determinants. These values are plotted in Figure 1. The remaining parts of equation (1) from Appendix S1were not computationally demanding. The value of 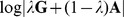 was computed just once and utilized for all traits being evaluated. Finally, drawing random values from the conditional (1) requires again linear interpolation from the 10,001 values.

### Data analysis

The method was evaluated with a real dataset consisting of a large sample of genotyped individuals provided by the EuroGenomics Consortium. In most genomic evaluation models used in animal breeding programs, genotyped sires are evaluated using their daughters' performance as phenotypes (usually known as the reference population or training set), and genome-enhanced prediction of young individuals (usually called prediction population) are made based on their genomic relationship with animals in the reference set.

An important question is the ability of the genome-based model at predicting the future performance of the progeny of these young sires. We set out a cross-validation scheme using the model above to check its predictive ability, and compared it with the model run at different values of lambda set *ad hoc*: 0. 01 (approximately equivalent to traditional BLUP using only genealogical information), 0.25, 0.50, 0.75 and 0.99 (the latter being approximately equivalent to G-BLUP using only genomic information).

#### Genomic and genealogical information

Data from 18,446 EuroGenomics progeny-tested sires was used in this study. The Bovine 50K chip (Illumina inc., San Diego) was used to genotype 54,609 SNPs in each sire. SNPs with an incidence of missing genotypes across individuals greater than 5%, or SNPs with minor allele frequency less than 5% were discarded, leaving 36,971 SNP for the analyses. After editing, 0.01% of the SNP genotypes were missing. These genotypes were then imputed with Beagle 3.3.2 (see Jiménez-Montero et al. [28] for more details). Pedigree data from 46,559 animals was used. This genealogical information included four generations of ancestors related to the genotyped individuals. In this study, the additive relationship matrix (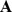) obtained from genealogical information was constructed using the algorithm of Meuwissen and Luo [29], which is implemented in the software Pedig [30]. In addition, the most often employed genomic relationship matrix (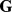) [5], [6] was also calculated. The genomic relationship between individuals *i* and *j* was calculated as
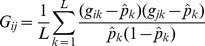
where 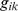 refers to the allele frequency value genotypes in *AA*, *Aa* and *aa*, coded as 1, 0.5 and 0, respectively, of individual *i* at locus *k* where *i* = 1, …, *n* and *k* = 1, …, *L*. The estimate of the allele frequency in the sample is denoted as 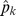. The two matrices (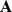 and 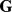) were combined into a kernel matrix (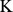), as described in the previous section.

#### Phenotypic information

Sire deregressed proofs (DRP) for five traits were used as phenotypic values. The traits included 3 production traits: milk yield (MY), fat yield (FY) and protein yield (PY), and 2 type traits: foot angle (FA) and udder depth (UD).

#### Training and testing data sets

The gain in predictive ability was assessed by cross-validation. Sires were divided into two groups, a training and a testing data set, according to year of birth. The January 2009 DRP was used as response variable in the training set, whereas, December 2011 DRP was used as a prediction target in the testing set. The testing data set included only sons of sires in the training set. This classification gave 14,487 training bulls born before 2005 and 3,959 testing bulls born after 2005. The minimum number of effective daughter contribution (EDC) allowed per sire was 20. Design of the training and testing data sets followed recommendations of Mäntysaari et al. [31].

In order to evaluate the behavior of the inferred weight on pedigree- and marker-based matrices, and also to assess the amount of information needed to improve genomic predictions, a random subset (*N* = 7,000) from the previously defined training data set was also employed as a reference population.

The model was implemented using a Bayesian approach via the Gibbs sampler. Each analysis was based on a chain of 25,000 iterations, with the first 10,000 iterations discarded as burn-in. There were 15,000 samples used for posterior inference, obtained by collecting each iteration from the chain following the burn-in.

#### Criteria for comparisons

The reliability of the genome-based predictions was assessed using the predicted direct genomic values (DGV) of bulls in the testing set and their December 2011 DRP. Three metrics were used: (1) Pearson's correlation coefficient, (2) the regression coefficient of the realized DRP on the estimated DGV, and (3) the mean squared error of predictions.

## Results and Discussion

**Figure 2:**
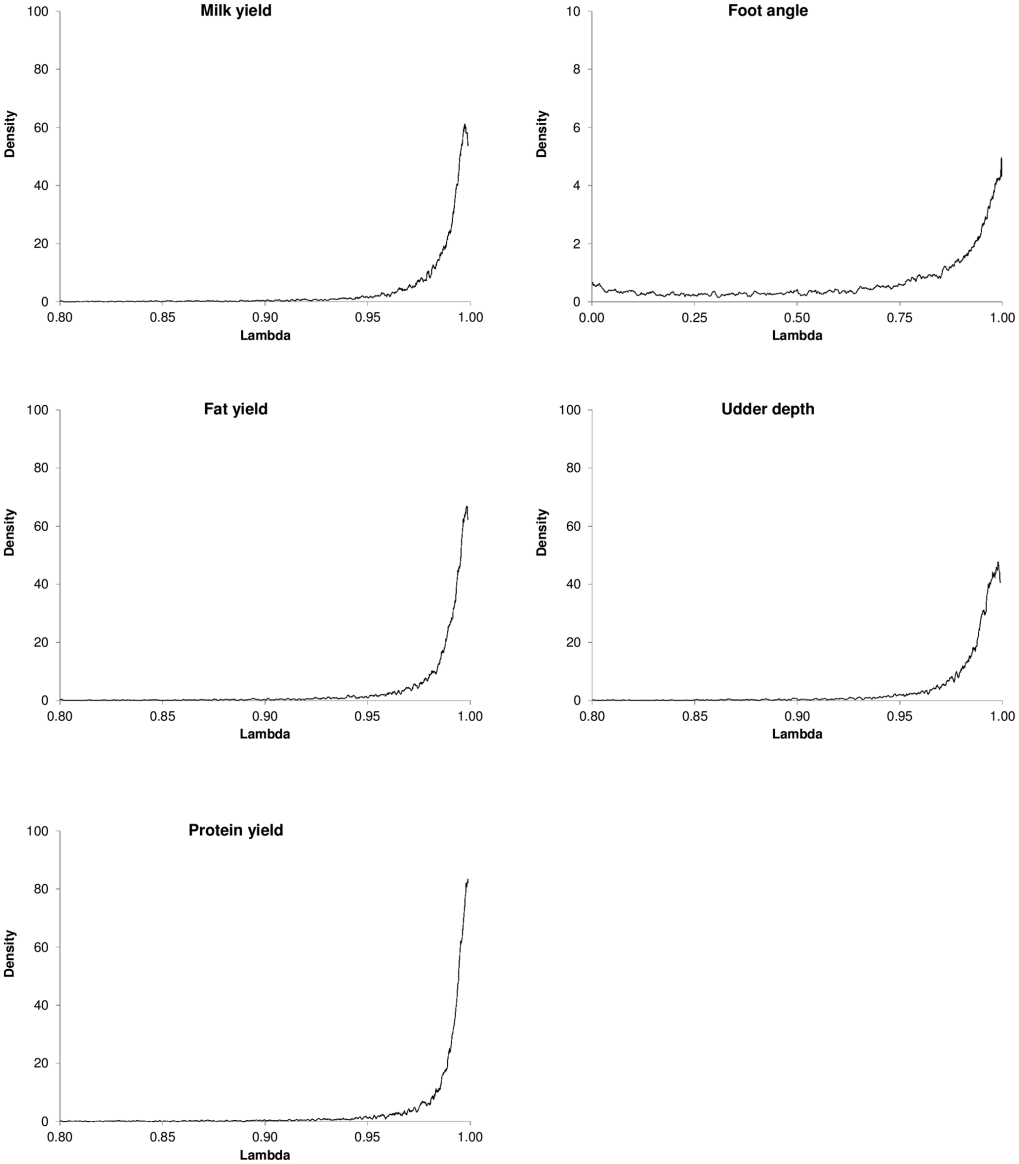
Posterior density of the inferred weight 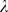
for the traits evaluated with *N* = 14,487. Notice the different scale for foot angle.

Figure 2 illustrates the large amount of uncertainly in the posterior density of the weight for the traits evaluated when *N* = 14,487. The posterior density of the inferred weight was very similar for most traits evaluated. The exception was FA, where a higher amount of uncertainly was detected, probably due to the smaller heritability of this trait. The posterior mean of the inferred weight (λ) also varied depending on the number of bulls in the training set and also with the trait evaluated (Table 2). When all available individuals were used as training set (*N* = 14,487) larger values of λ were obtained, given more weight to the genomic information than in the situation where only 7,000 bulls were used in the reference data set. In addition, the emphasis placed on genomic information was larger in production traits than in type traits, suggesting that most of the additive genetic variability in production traits was captured by genomic relationships. Posterior means of λ for FA were much lower, possible due to low heritability of the trait.

**Table 2:** Posterior mean of the inferred lambda for each trait evaluated and reference population size.

	Reference population size	
Trait	7,000	14,487
Milk yield	0.92	0.96
Fat yield	0.93	0.96
Protein yield	0.97	0.98
Foot angle	0.62	0.72
Udder depth	0.92	0.95

### Correlation

**Figure 3:**
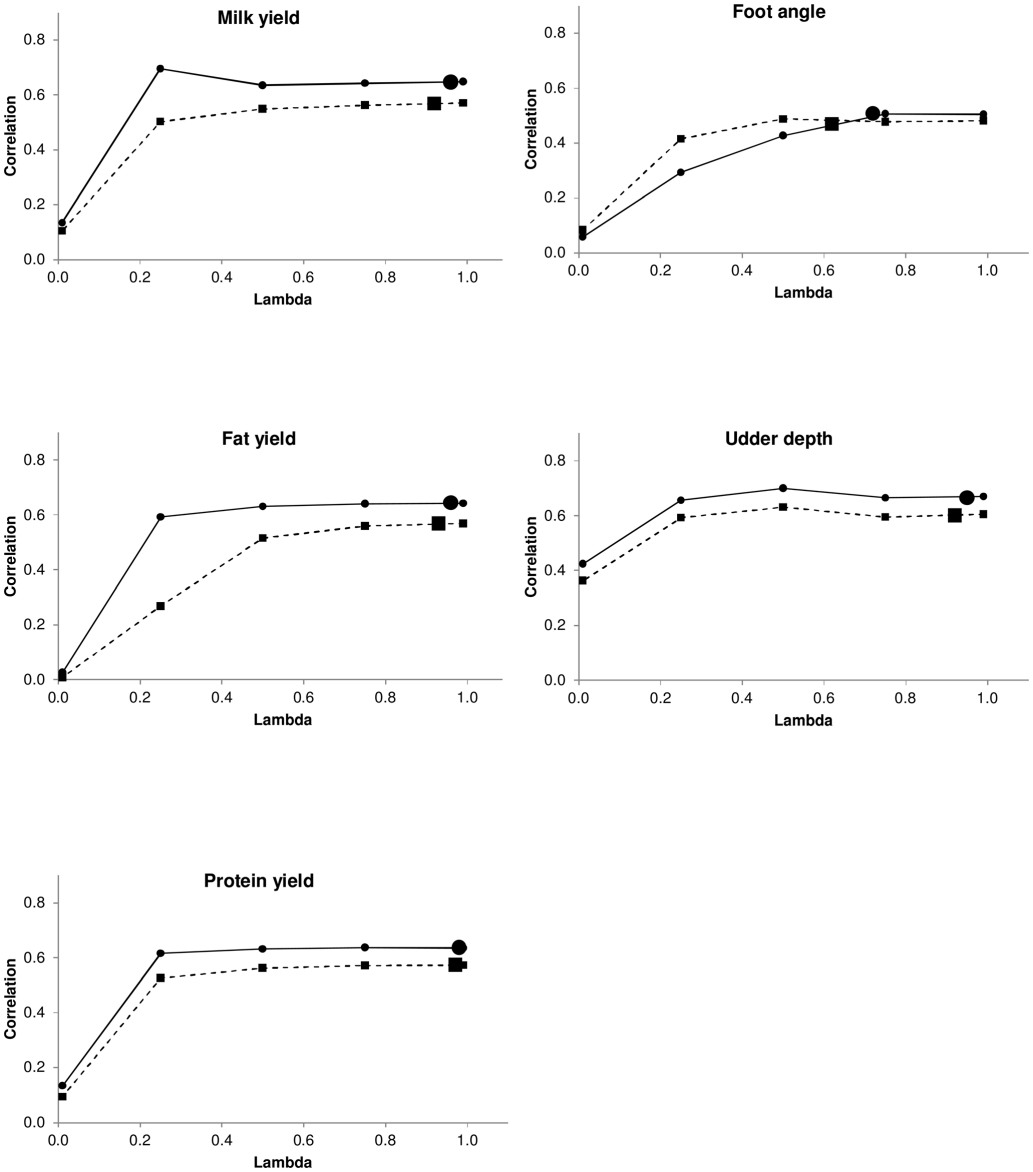
Correlation of predictions. Dashed lines and small squares represent fixed weights with *N* = 7,000. Solid lines and small circles represent fixed weights with *N* = 14,487. The large square represents the sampled weight for *N* = 7,000. The large circle represents the sampled weight for *N* = 14,487.

Correlation coefficients when 7,000 or 14,487 bulls were used as training data set are shown in Figure 3. In general, the trend was similar for all traits evaluated, with longer predictive correlation when the training sample was 14,487, as expected. When a larger weight was given to the additive relationship matrix (λ close to 0) the correlation coefficient was lower than in situations where more weight was given to genomic information (λ close to 1). In addition, the predictive correlation was virtually insensitive with respect to λ values between 0.5 and 1.

As noted above, the correlation coefficient with *N* = 14,487 was, in general, larger in all situations than the correlations obtained with the smaller data set (except for FA and λ = 0.01, 0.25 and 0.5). For instance, the predictive correlation for UD when λ was fixed to 0.75 was 0.59 and 0.66 for *N* = 7,000 and *N* = 14,487, respectively. Fixed λ values did not lead to DGV predictions with larger accuracy in the validation set (e. g., milk yield or udder depth), because it provided larger bias in the predictions, with larger mean squared error estimates, as shown below.

### Over and under predictions

**Figure 4:**
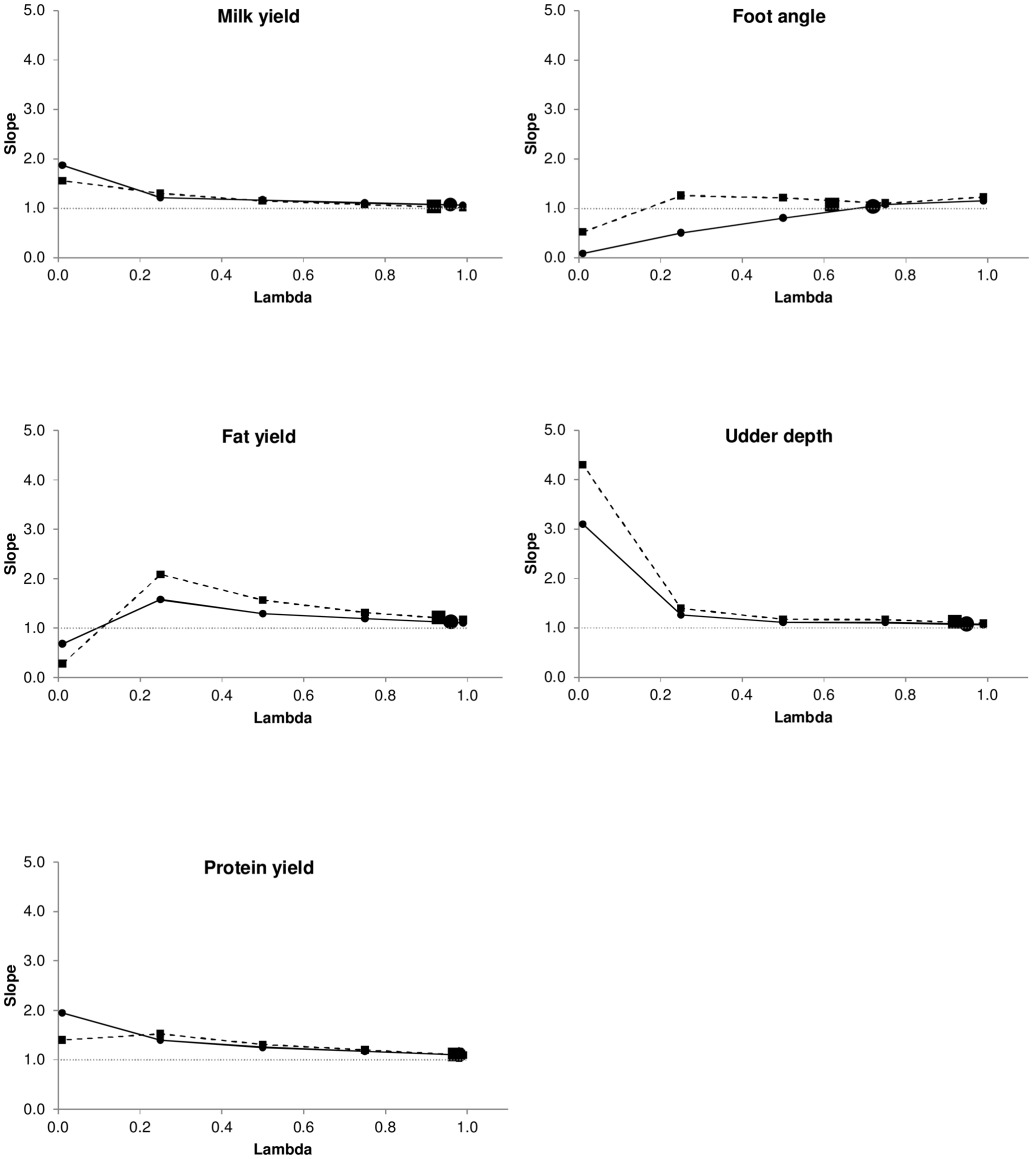
Slope of predictions. Dashed lines and small squares represent fixed weights with *N* = 7,000. Solid lines and small circles represent fixed weights with *N* = 14,487. The large square represents the sampled weight for *N* = 7,000. The large circle represents the sampled weight for *N* = 14,487. The optimum value is one.

A regression coefficient of DRP in the testing set on genomic predictions lower than one indicates an overestimation, whereas a coefficient larger than one indicates an underestimation of the target trait. Figure 4 shows the regression coefficients when 7,000 (dashed lines and squares) or 14,487 (solid lines and circles) bulls were used in the training data sets. Most regressions were close to one, matching variations in predicted and observed values. The exceptions were FY and FA for λ = 0.01 for *N* = 7,000, FY for λ = 0.01 and FA and λ = 0.01, 0.25 and 0.5 for *N* = 14,487. In addition, as λ increased the slope was nearly one. Likewise, the sampled values of λ (large square and circle) were very close to one, *i. e*., when more weight was given to the genomic information.

Results obtained with *N* = 14,487 bulls in the training data set were analogous to those obtained with a reduced data set. However, with more individuals in the training data set, the regression coefficients obtained at different λ values were closer to one than in the situation with *N* = 7,000 individuals.

### Mean squared error

**Figure 5:**
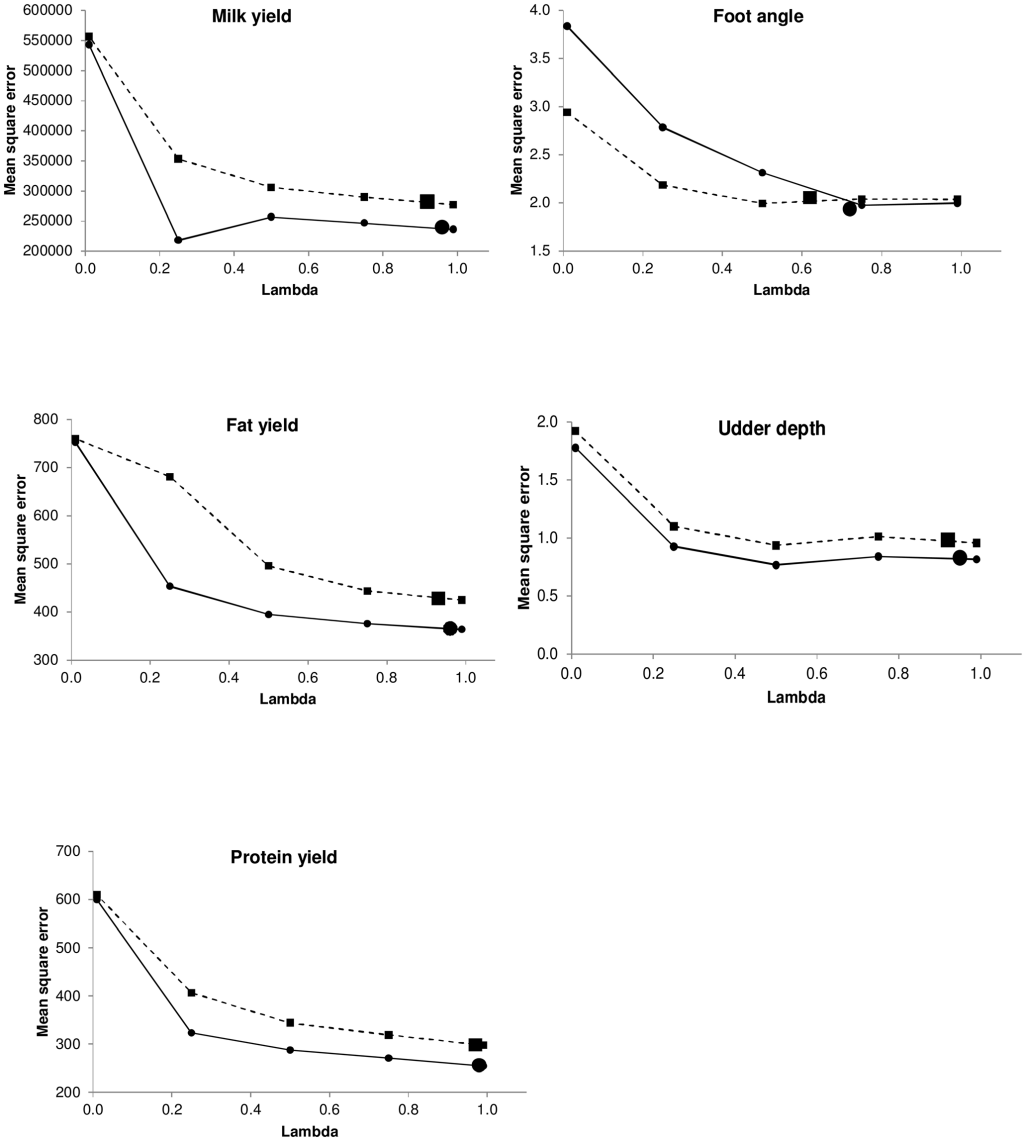
Mean squared error of predictions. Dashed lines and small squares represent fixed weights with *N* = 7,000. Solid lines and small circles represent fixed weights with *N* = 14,487. The large square represents the sampled weight for *N* = 7,000. The large circle represents the sampled weight for *N* = 14,487.

The behavior of the mean squared error (Figure 5) for *N* = 7,000 and *N* = 14,487 was similar to the behavior of the correlation and regression coefficients, that is, more weight to the genomic information provided a smaller mean squared error. The percentage of mean squared error reduction between λ = 0.01 (mostly pedigree information) and the estimated λ ranged between 30% and 51% for *N* = 7,000, and between 50% and 57% for *N* = 14,487. The percentage change in mean squared error from using the estimated λ and λ = 0.99 (mostly genomic information) ranged between a reduction of 3% and an increase of 2%.

In addition, use of more individuals induced a smaller mean squared error, and the reduction from 7,000 to 14,487 bulls was 6%–15% across traits. Interestingly, when more weight was given to genealogical information, differences between the mean squared error for *N* = 7,000 and *N* = 14,487 were smaller than when more weight was given to genomic information. This behavior was also detected for the correlation (Figure 3) but it was subtler for the regression coefficient (Figure 4). Therefore, differences in mean squared error across different fixed λ values (Figure 5) were higher than differences in correlation across different fixed λ (Figure 3), favoring the mean squared error as a criterion to discriminate between models.

Liu et al. [32] investigated the impact of including an *ad hoc* weight to infinitesimal effects on genomic predictions. These authors used three different percentages (5%, 10% and 15%) of infinitesimal variance relative to the total genetic variance. They found that, for traits with larger heritability, e. g. production traits, somatic cell score, stature and rump angle, the optimal weights assigned to the infinitesimal variance appeared to be less than 5%. For the conformation traits as rump width and body conditional score, values of 10% or higher were “optimal”. These authors also indicated that the optimal weight differed among traits. Therefore, trait specific weighting factors should be used in single step blending methods and in G-BLUP models with an infinitesimal effect. Gao et al. [33] indicate that a weighting factor of 0.40 reduced “bias”, and weighting factors around 0.15–0.20 gave the highest reliability.

Haile-Mariam et al. [34] estimated the proportion of variance in daughter trait deviations of dairy bulls explained by 45,993 SNPs for 29 traits in Australian Holstein-Friesian dairy cattle. They compared these proportions to the proportion of variance in daughter trait deviations explained by the additive relationship matrix derived from the pedigree, as well as by the sum of variances explained by both pedigree and marker information when these were fitted simultaneously. Their results suggested that the Bovine SNP50 array, widely used for genomic evaluations in dairy cattle, does not capture 100% of the additive genetic variation for 29 traits, with a range of 90% for milk yield to 32% for fertility. When fitting genomic and pedigree relationships simultaneously, the residual variance in daughter trait deviations was smaller than when fitting either source of information individually. They indicated that the proportion of genetic variance accounted for by the genomic relationships could be used to modify the blending equations used to calculate genomic estimated breeding values from direct genomic breeding values and parent average. Their results, from a validation population of young dairy bulls, suggest that this modification can improve reliability of genomic estimated breeding values by up to 5%.

Further research could be done with two different models where the 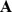 and 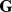 matrices are fitted as single kernels. Results from a model fitting 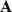 matrix alone for MY and *N* = 14,487 indicated that the correlation, slope and mean squared error were 0.06, 0.09 and 712,763, respectively. Accordingly, results for λ = 0.01 were 0.13, 1.87 and 543,066 for the correlation, slope and mean squared error respectively. Results for the remaining traits and *N* = 7,000 where equivalent to those for MY and *N* = 14,487 (data not shown). Regarding fitting 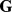 alone, several methods have been proposed to make inferences with the genomic matrix positive definite [35]. The mixed model equations can be modified to cope with non-singular relationship matrices or the non-singular relationship matrices can be transformed into positive definite by adding a positive number to their diagonal. However, it is expected that results will be very similar to those showed for λ = 0.99.

A multi-kernel approach was proposed by De los Campos et al. [23] with 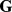 and 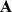 acting as two different kernels with corresponding variance components. The multi-kernel approach and that described herein are expected to lead to equivalent models if the ‘true’ value of lambda is used, as described in García-Cortés and Toro [36]. Nonetheless, further research could be done to compare the method proposed in the present study and the multi-kernel approach proposed by De los Campos et al. [23].

## Conclusions

This study proposed and evaluated a semi-parametric Bayesian method for combining marker- and pedigree-based information in genomic predictions involving a large reference population. The advantage of using both sources of information is that QTL effects not captured by SNP effects might be captured by a parental average or by infinitesimal additive effects, especially if the population genotyped is not large enough.

Results indicated that production traits showed a larger influence from genomic information than type traits. This could be related to heritability of the trait, because larger estimates of λ were associated with larger values of heritability (*h^2^* = 0.28, 0.28, 0.28, 0.12 and 0.30 for MY, FY, PY, FA and UD, respectively). The heritability is relevant in genomic predictions, the lower heritability the larger number of records is required in the reference population to achieve high accuracies of GEBV in unphenotyped animals. Our results indicated a greater accuracy of genomic predictions when using 14,487 bulls than when 7,000 randomly chosen bulls were employed as reference population. The reason is that, when more phenotypic records are available, unknown parameters are better estimated, leading to a larger accuracy of genomic selection. In dairy cattle, gains from genomic selection are seemingly larger for production than for fertility traits, in part due to the lower accuracy of estimated breeding values for fertility. The inferred weight also depends on the number of bulls in the training set. A higher number of bulls in the reference population produced a larger value of λ, giving more weight to genomic information. In addition, the emphasis placed on genomic information was larger in production traits than in type traits, suggesting that most of the additive genetic variability in production traits can be captured by genomic relationships.

We have shown that fitting a weight on pedigree- and marker-based information in a genome-enabled prediction model is beneficial. The optimal weight differs between traits. The size of the reference population is an important factor affecting the accuracy of genome-based predictions. Our study has also shown that SNP based evaluation is more efficient than standard genetic evaluation based on pedigree.

## Supporting Information

Appendix S1 **Implementation of a hierarchical Bayesian model for inferring model unknowns.**
(DOC)
