# Supplementary material for: Combining Genomic and Genealogical Information in a Reproducing Kernel Hilbert Spaces Regression Model for Genome-Enabled Predictions in Dairy Cattle
Source: PLoS One. 2014 Mar 26;9(3):e93424. doi: 10.1371/journal.pone.0093424 (PMC3966896; doi:10.1371/journal.pone.0093424)
Supplement: Appendix S1 — Implementation of a hierarchical Bayesian model for inferring model unknowns. (DOC) [file pone.0093424.s001.doc]

**Appendix S1**

Hierarchical Bayesian model for inferring model unknowns

1) Likelihood: $p(\mathbf{y}|\beta,\alpha,\lambda,\sigma_{e}^{2})=\prod_{i=1}^{n} N(y_{i}|\mathbf{x}_{i}^{'}\beta+\mathbf{k}_{i}\alpha,\sigma_{e}^{2})$

2) Joint Prior:

$p(\beta,\alpha,\lambda,\sigma_{\alpha}^{2},\sigma_{e}^{2})=p(\beta|\sigma_{\beta}^{2})p(\alpha|\lambda,\sigma_{\alpha}^{2})p(\lambda)p(\sigma_{\alpha}^{2})p(\sigma_{e}^{2})$

$=N(\beta|0,\mathbf{I}\sigma_{\beta}^{2})N(\alpha|0,\mathbf{K}^{-1}\sigma_{\alpha}^{2})$

$\times\chi^{-2}(\sigma_{\beta}^{2}|d_{\beta},S_{\beta})\chi^{-2}(\sigma_{\alpha}^{2}|d_{u},S_{u})\chi^{-2}(\sigma_{e}^{2}|d_{e},S_{e})p(\lambda)$

Above, $n$ is the number of records in the training set, $N(y_{i}|\mathbf{x}_{i}^{'}\beta+\mathbf{k}_{i}\alpha,\sigma_{e}^{2})$, $N(\beta|0,\mathbf{I}\sigma_{\beta}^{2})$ and $N(\alpha|0,\mathbf{K}^{-1}\sigma_{\alpha}^{2})$ are normal densities centered at $\mathbf{x}_{i}^{'}\beta+\mathbf{k}_{i}\mathbf{\alpha}$, or 0, with variances or covariance matrices $\sigma_{\beta}^{2}$, $\mathbf{K}^{-1}\sigma_{\alpha}^{2}$ and $\sigma_{e}^{2}$, respectively; $\chi^{-2}(\sigma_{\beta}^{2}|d_{\beta},S_{\beta})$, $\chi^{-2}(\sigma_{\alpha}^{2}|d_{\alpha},S_{\alpha})$ and $\chi^{-2}(\sigma_{e}^{2}|d_{e},S_{e})$are scaled-inverted chi-square densities, with $d_{\bullet}$ degrees of freedom and scale $S_{\bullet}$.

3) Joint posterior distribution:

$p(\beta,\alpha,\lambda,\sigma_{\alpha}^{2},\sigma_{e}^{2}|\mathbf{y})=\prod_{i=1}^{n} N(y_{i}|\mathbf{x}_{i}^{'}\beta+\mathbf{k}_{i}^{'}\alpha,\sigma_{e}^{2})$

$\times N(\beta|0,\mathbf{I}\sigma_{\beta}^{2})N(\alpha|0,\mathbf{K}^{-1}\sigma_{\alpha}^{2})$

$\times\chi^{-2}(\sigma_{\beta}^{2}|d_{\beta},S_{\beta})\chi^{-2}(\sigma_{\alpha}^{2}|d_{u},S_{u})\chi^{-2}(\sigma_{e}^{2}|d_{e},S_{e})p(\lambda)$

The full conditional distribution of any unknown is obtained by removing from the right-hand side of the equation above the components that do not involve such an unknown. The remaining components are kernels of known distribution given conjugate priors were chosen. In this model, all full conditionals have closed form except that of $\lambda$**.**

4) Full conditional distributions:

a) Regression coefficients for nuisance effects ($\beta$)

$p(\beta_{j}|else)\propto\left[\prod_{i=1}^{n} N(y_{i}|\mathbf{x}_{i}^{'}\beta+\mathbf{k}_{i}^{'}\alpha,\sigma_{e}^{2})\right]\times N(\beta_{j}|0,\sigma_{\beta}^{2})$

$\propto\left[\prod_{i=1}^{n} N(y_{i}^{*}|\mathbf{x}_{i}\beta_{j},\sigma_{e}^{2})\right]\times N(\beta_{j}|0,\sigma_{\beta}^{2})$

This is the kernel of a normal distribution with mean equal to the solution of $\hat{\beta}_{j}=\sigma_{e}^{-2}\sum_{i=1}^{n} x_{ij}y_{i}^{*}$, where $y_{i}^{*}=y_{i}-\sum_{p\neq j} x_{ip}\beta_{p}-\mathbf{k}_{i}\alpha$. The variance is $\left(\sigma_{e}^{-2}\sum_{i=1}^{n} x_{ij}^{2}+\sigma_{\beta}^{-2}\right)^{-1}$**.** In practice one can set $\sigma_{\beta}^{2}$ large enough so that an effectively flat prior is assigned to these coefficients.

b) Non-parametric coefficients ($\alpha$)

$p(\alpha_{j}|else)\propto\left[\prod_{i=1}^{n} N(y_{i}|\mathbf{x}_{i}^{'}\beta+\mathbf{k}_{i}^{'}\mathbf{\alpha},\sigma_{e}^{2})\right]\times N(\alpha_{j}|0,\mathbf{K}^{-1}\sigma_{\alpha}^{2})$

$\propto\left[\prod_{i=1}^{n} N(y_{i}^{**}|\mathbf{k}_{i}^{'}\alpha_{j},\sigma_{e}^{2})\right]\times N(\alpha_{j}|0,\mathbf{K}^{-1}\sigma_{\alpha}^{2})$

This is the kernel of a normal distribution with mean equal to the solution of $\hat{\alpha}_{j}=\sigma_{e}^{-2}\sum_{i=1}^{n} k_{ij}y_{i}^{**}$, where $y_{i}^{**}=y_{i}-\mathbf{x}\beta-\sum_{p\neq j} k_{ip}\alpha_{p}$; and variance $\left(\sigma_{e}^{-2}\sum_{i=1}^{n} k_{ij}^{2}+\sigma_{\alpha}^{-2}\right)^{-1}$**.**

c) Kernel variance ($\sigma_{\alpha}^{2}$)

Given that $\mathbf{K}\sigma_{\alpha}^{2}=[\lambda\mathbf{G}+(1-\lambda)\mathbf{A}]\sigma_{\alpha}^{2}$

$p(\sigma_{\alpha}^{2}|else)\propto N(\alpha|0,\mathbf{K}^{-1}\sigma_{\alpha}^{2})\times\chi^{-2}(\sigma_{\alpha}^{2}|d_{\alpha},S_{\alpha})$

$\propto\chi^{-2}(\sigma_{\alpha}^{2}|d.f.=d_{\alpha}+n,S=S_{\alpha}+\alpha'\mathbf{K}\alpha)$

where $n$ is the order of the square matrix $\mathbf{K}$.

d) Residual variance ($\sigma_{e}^{2}$)

$p(\sigma_{e}^{2}|else)\propto\left[\prod_{i=1}^{n} N(y_{i}|\mathbf{x}_{i}^{'}\beta+\mathbf{k}_{i}^{'}\mathbf{\alpha},\sigma_{e}^{2})\right]\times\chi^{-2}(\sigma_{e}^{2}|d_{e},S_{e})$

$\propto\chi^{-2}(\sigma_{e}^{2}|d.f.=d_{e}+n,S=S_{e}+\mathbf{e}'\mathbf{e})$

where $\mathbf{e}=\mathbf{y}-\mathbf{x}_{i}^{'}\beta-\mathbf{k}_{i}^{'}\mathbf{\alpha}$.

e) Weighting factor for the kernel matrix ($\lambda$)

Here, an inverse function [1] was used as proposed for drawing samples from the conditional distribution, which does not have a closed form. The probability density function to evaluate is

$p(\lambda|else)\propto\left[\prod_{i=1}^{n} N(y_{i}|\mathbf{x}_{i}^{'}\beta+\mathbf{k}_{i}^{'}\mathbf{\alpha},\sigma_{e}^{2})\right]\times N(\mathbf{\alpha}|\mathbf{K}^{-1}\sigma_{\alpha}^{2})p(\lambda)$

$\propto\exp\left[-\frac{(y-\mathbf{X}\beta-\mathbf{K\alpha})^{2}}{2\sigma_{e}^{2}}\right]\frac{1}{\left|\mathbf{K}\right|^{\frac{1}{2}}}\exp\left[-\frac{\mathbf{\alpha}'\mathbf{K\alpha}}{2\sigma_{\alpha}^{2}}\right]$

Taking logarithms from the above function we obtain:

$\log(p(\lambda|else))\propto-\frac{1}{2\sigma_{e}^{2}}[\mathbf{y}-\mathbf{X}\beta-(\lambda\mathbf{G}+(1-\lambda)\mathbf{A})\mathbf{\alpha}]^{'}[\mathbf{y}-\mathbf{X}\beta-(\lambda\mathbf{G}+(1-\lambda)\mathbf{A})\mathbf{\alpha}]$

$-\frac{1}{2}\log\left|\lambda\mathbf{G}+(1-\lambda)\mathbf{A}\right|-\frac{1}{2\sigma_{\alpha}^{2}}\mathbf{\alpha}'(\lambda\mathbf{G}+(1-\lambda)\mathbf{A})\mathbf{\alpha}$

$=-\frac{1}{2\sigma_{e}^{2}}[(\mathbf{y}-\mathbf{X}\beta-\mathbf{A\alpha})-\lambda(\mathbf{G}-\mathbf{A})\mathbf{\alpha}]^{'}[\mathbf{y}-\mathbf{X}\beta-(\lambda\mathbf{G}+(1-\lambda)\mathbf{A})\mathbf{\alpha}]$

$-\frac{1}{2}\log\left|\lambda\mathbf{G}+(1-\lambda)\mathbf{A}\right|-\frac{1}{2\sigma_{\alpha}^{2}}\mathbf{\alpha}'\mathbf{G\alpha}\lambda-\frac{1}{2\sigma_{\alpha}^{2}}\mathbf{\alpha}'\mathbf{A\alpha}(1-\lambda)$ (1)

This simplifies computational requirements because $(\mathbf{y}-\mathbf{X}\beta-\mathbf{A\alpha})$ may be calculated using the residual updates[2], $(\mathbf{G}-\mathbf{A})$ can be stored in memory at the beginning of the algorithm and, $\mathbf{\alpha}'\mathbf{G\alpha}$ and $\mathbf{\alpha}'\mathbf{A\alpha}$ are quadratic forms that can be calculated in a relatively fast fashion.

**References**

1. Devroye L (1986) Non-Uniform random variate generation. New York: Springer-Verlag.

2. Legarra A, Misztal I (2008) Genome-wide selection computing strategies. J Dairy Sci 91: 360–366.
